# Concentration of Povidone-Iodine Pocket Irrigation in Implant-Based Breast Surgery: A Scoping Review

**DOI:** 10.1007/s00266-025-04660-y

**Published:** 2025-01-23

**Authors:** Ojochonu D Anthony, Ishith Seth, Warren M Rozen

**Affiliations:** 1 Faculty of Medicine, Nursing and Health Sciences, Monash University, Melbourne, Australia; 2 Department of Plastic, Reconstructive and Hand Surgery, Peninsula Health, Frankston, VIC Australia; 3 Department of Plastic and Reconstructive Surgery, Peninsula Clinical School, Central Clinical School, Faculty of Medicine, Monash University, 2 Hastings Road, Frankston, VIC 3199 Australia

**Keywords:** Povidone-iodine, Betadine, Breast implant, Breast augmentation, Breast reconstruction, Capsular contracture

## Abstract

**Background:**

In implant-based breast surgery, microbial contamination of implant surfaces predisposes complications such as overt periprosthetic infection and has been linked to capsular contracture (CC). Anti-microbial practices, including povidone-iodine (PVP-I) breast pocket irrigation, are routinely employed to minimise these risks. No standardised protocol for using this antiseptic exists, particularly concerning the ideal concentration. This review investigates how PVP-I concentration affects outcomes in these procedures while highlighting research gaps.

**Methods:**

Using PRISMA-ScR guidelines, a systematic search was conducted across MEDLINE, Embase, Scopus, and PubMed databases from their inception to June 2024. Studies were screened using pre-determined criteria for inclusion. The methodological quality of relevant studies was assessed using the MINORS tool. Data regarding basic characteristics, PVP-I irrigation implementation, and outcomes (primarily periprosthetic infection and CC) were extracted for analysis.

**Results:**

Nine articles, primarily observational studies, and retrospective analyses were included. These mainly focused on breast augmentation with a few including reconstruction. There was considerable heterogeneity in surgical techniques, and reported PVP-I concentrations ranged from 4 to 20%. This was further confounded by frequent mixing of irrigation solution with antibiotics. Although infection and CC rates were frequently reported, most studies did not specify outcome data for patients receiving PVP-I irrigation.

**Conclusions:**

While PVP-I irrigation is extensively used in implant surgeries, the current evidence base is insufficient to determine the optimal concentration and application techniques. This review underscores the need for further detailed research to establish evidence-based guidelines for PVP-I use, aiming to improve patient care and surgical outcomes in breast surgery.

**Level of Evidence III:**

This journal requires that authors assign a level of evidence to each article. For a full description of these Evidence-Based Medicine ratings, please refer to the Table of Contents or the online Instructions to Authors www.springer.com/00266.

## Introduction

Implant-based breast surgeries, encompassing both cosmetic and reconstructive procedures, are among the most frequently performed operations in plastic surgery worldwide [1–5]. These transform lives by allowing individuals to modify or restore their breast contour and volume [6–9]. The risk of breast implant surface contamination with microorganisms during insertion is a significant concern, potentially leading to a range of postoperative complications [10]. Firstly, it can progress to overt periprosthetic infections which often require further surgical intervention [11, 12]. Another common issue, capsular contracture (CC), causes considerable patient and surgeon dissatisfaction and is a leading cause for corrective re-operations [13–15]. Subclinical infections are believed to contribute to the development of CC by triggering an immune response that leads to chronic inflammation [12, 16–18].

Povidone-iodine (PVP-I), a well-established antimicrobial agent, offers many benefits for antisepsis and wound healing [19–21]. Its key characteristics include broad-spectrum antimicrobial efficacy, effective biofilm penetration, and a notable lack of resistance development, all while being well tolerated by patients [22–26]. Stringent antimicrobial protocols, including the use of PVP-I for breast pocket irrigation and implant immersion as suggested in the widely adopted “14-point plan,” have shown potential in reducing peri-implant infection and CC incidence [27–31]. PVP-I has been employed in this context as a standalone agent or in combination with other antimicrobials [32–36]. Although an estimated one-third of surgeons worldwide reportedly use PVP-I in some capacity, [37] there is significant variation in concentrations used, ranging from as low as 4% to as high as 20% [32, 35, 38, 39]. This variability prompts questions about the optimal concentration for maximising efficacy and minimising potential adverse effects, underscoring a notable gap in the literature. Bridging this gap is essential for improving antimicrobial prophylaxis in implant-based breast surgeries and enhancing patient outcomes.

This scoping review aims to consolidate and analyse research on the role of povidone-iodine in breast pocket irrigation and prosthesis immersion during implant-based breast surgeries. Focusing on the antiseptic*’*s concentration, it seeks to identify an optimal balance of safety and effectiveness. Key areas of interest include the incidence of periprosthetic breast infection and CC, as well as other outcomes like implant deflation or rupture. The findings may enhance the evidence-based standardisation of antimicrobial practices in breast surgery, influencing future clinical guidelines.

## Methods

This method was formulated using the PRISMA-ScR guidelines [40]. A systematic literature search was conducted across the MEDLINE, Embase, Scopus and PubMed databases from their inception to June 2024. The search strategy was meticulously designed to ensure consistency across databases, focusing on titles and abstracts.

The search terms employed were: (breast OR mammary) AND (povidone OR iodine OR povidone-iodine OR iodo-povidone OR betadine OR antiseptic OR irrigat* OR lavage OR immers* OR soak*) AND (implant* OR prosthe* OR endoprosthe* OR pocket).

The reference lists of relevant articles were investigated for additional studies to enhance the scope of evidence. The Covidence systematic review platform [41] was utilised to assess studies, beginning with an initial screening based on titles and abstracts, followed by a full-text analysis to determine final inclusion or exclusion.

Two authors (ODA and IS) were responsible for the identification, screening, and inclusion of studies, and the third author addressed any issues related to eligibility. The inclusion criteria were the following: human patient studies on implant-based cosmetic or reconstructive breast procedures using povidone-iodine for implant immersion or breast pocket irrigation, alone or with other antimicrobials. Studies reporting outcomes such as periprosthetic infection, capsular contracture, or implant-associated anaplastic large cell lymphoma. The included study designs were randomised controlled trials, prospective and retrospective comparative studies as well as observational studies.

The exclusion criteria were the following: studies not incorporating povidone-iodine in breast pocket irrigation or with unspecified solution components. Articles involving transgender patients undergoing gender-affirming surgery or paediatric patients. In-vitro studies and studies involving non-human participants. Non-English language publications and inaccessible full-text articles. Additionally, given the significant advancements and stabilisation in breast surgery techniques and implants in the last few decades, publications dated before the year 2000 were also excluded.

### Quality Assessment

The methodologic index for non-randomised studies (MINORS) scale was used to appraise the methodological quality of the included non-randomised studies [42]. Each parameter on the scale is scored from zero (aspect not reported) to two (aspect reported and adequate). Non-comparative studies are scored using eight parameters, with a maximal score of 16. For comparative studies, four parameters are added to a total of 12 with a maximum score of 24.

For this review, it was determined that in non-comparative studies, a score of 8 or below indicated poor quality, 9–14 signified moderate quality, and 15–16 denoted high quality. For comparative studies, the quality benchmarks were set at 14 or below for poor, 15–22 for moderate, and 23–24 for high quality. The scoring was conducted by the two authors (ODA and IS) and validated by the senior author (WMR).

### Data Extraction

The background characteristics of the included studies were noted; these included:

- Study design
- Total number of patients
- Number/proportion of patients receiving cosmetic or reconstructive procedures
- Number of implants inserted
- Details about incision type and implant placement
- Types, textures and shapes of implants

Further, pertinent intervention and outcome details were extracted for the analysis such as:

- Proportion of patients receiving povidone-iodine treatment
- Concentration of povidone-iodine used
- Additional antimicrobials used in solution
- Mean follow-up period
- Rate of periprosthetic breast infection
- Rate of CC and criteria used to determine
- Rate of other relevant outcomes

### Statistical Analysis

Considerable heterogeneity was observed in the collected articles, particularly regarding study designs, patient demographics, surgical methods, and outcome measurements. This variability in research parameters indicated that a conventional statistical analysis, including attempts at result aggregation, would be neither practical nor significant. As a result, and also due to the absence of high-evidence studies in this area, a qualitative analysis approach was adopted instead of a quantitative statistical one. This method allowed for a more nuanced interpretation of the findings without the constraints of statistical homogenisation.

## Results

**Fig. 1:**
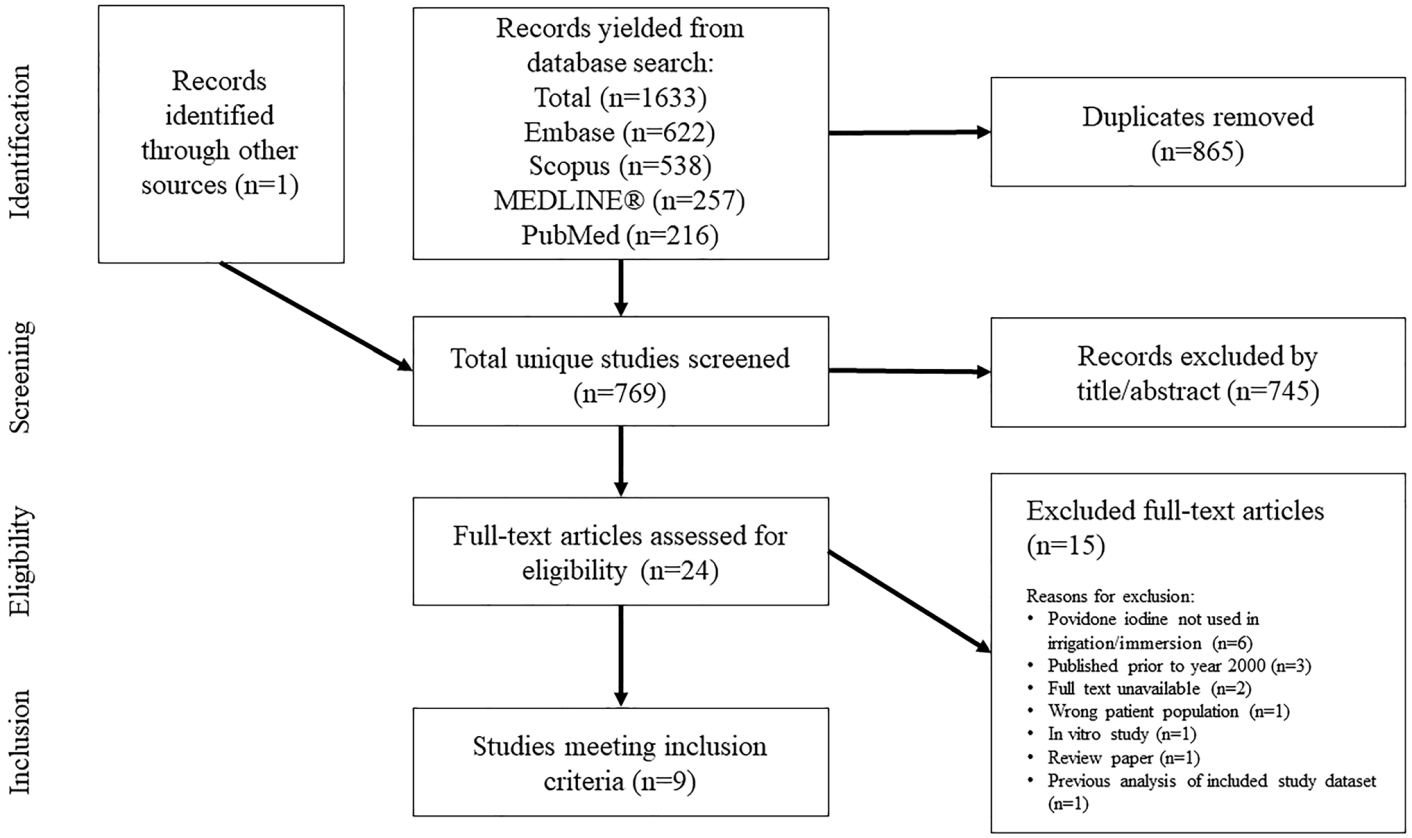
PRISMA flowchart illustrating the identification and screening process of studies for inclusion. Reporting checklist: The authors have completed the PRISMA-ScR reporting checklist

A total of 1634 records were identified, with 769 remaining after duplicates were removed. Following a title and abstract screening, 24 articles underwent full-text review, resulting in the inclusion of nine studies [32–36, 39, 43–45] that met all criteria for relevance (Fig. 1). Exclusions were largely due to studies lacking PVP-I in their irrigation solutions, in vitro studies, and articles focused on gender-affirming surgery. Two studies were excluded because their full texts were unavailable.

### Characteristics of Studies

As presented in Table 1, the studies were published between 2006 and 2022 and were primarily conducted in the USA. Study designs included prospective observational cohort studies and retrospective analyses of records. Sample sizes ranged from 143 to 17,656 patients. Six studies focused solely on cosmetic procedures, with three including cosmetic and reconstructive surgeries. Considerable heterogeneity was observed in the reporting of surgical techniques (primarily incision sites and implant placement) as well as the implant material, texture and shape.

**Table 1:** Characteristics of included studies

Study (Author, year)	Country	Study design (comparative nature)	MINORS score	Total number of patients	Operation type	Number and types of implants	Incision and implant location
Adams et al. (2006) [32]	USA	Prospective observational study (N)	12/16	250	Cosmetic (*n * = 187) PBA (*n * = 165) AM (*n * = 22)	Total (*n * = 374) Saline (*n *= 290; 228 smooth, 62 textured) Silicone gel (*n *= 84; 84 textured)	Incision: NR Placement (% cosmetic patients): Dual plane 1 (77%) Dual plane 2 (15.5%) Dual plane 3 (6.4%) Subpectoral (0.5%) Subglandular (0.5%)
					Reconstructive; BR (*n *= 63)	Total (* n *= 99) Saline (*n *= 15; 8 smooth, 7 textured) Silicone gel (*n *= 84; 44 smooth, 40 textured)	Incision: NR Placement: NR
Araco et al. (2007) [43]	United Kingdom	Retrospective analysis (N)	12/16	3002	Cosmetic: PBA (*n *= 2824) AM (*n* = 178)	Total number of implants NR All textured silicone gel Shape (% patients):- round (90%)- anatomical (10%)	Incision: Inframammary (*n *= 2824; BA group) Peri-areolar (*n *= 178; A-M group) Placement: Subglandular (*n *= 750) Subfascial (*n *= 752) Subpectoral (*n *= 1000) Dual plane (*n *= 500)
Blount et al. (2013) [33]	USA	Retrospective analysis (N)	9/16	856	Cosmetic: PBA	Total number of implants NR Silicone gel (53%) Saline (47%) Textured (21%) Smooth (75%) *(% of patients)	Incision: Inframammary (81.9%) Trans-axillary(16.8%) Placement: Subglandular (0.6%) Subpectoral (99.4%)
Calobrace et al. (2018) [34]	USA	Prospective observational study (N)	11/16	2565	Cosmetic: PBA	Total (*n *= 5122; smooth 62%, textured 38%) All implants were silicone gel and round in shape	Incision (% devices): Peri-areolar (29%) Inframammary (71%) Placement: Subglandular (56%) Subpectoral (44%)
Giordano et al. (2013) [35]	Finland	Retrospective analysis (C)	18/24	330	Cosmetic: PBA	Total (n = 660) All implants were silicone gel, textured and anatomical in shape	Incision: Inframammary (100%) Placement: Dual plane (100%)
McGuire et al. (2017) [44]	USA	Prospective observational study (N)	11/16	17,656	Cosmetic (*n *= 7691) PBA (*n *= 5059) RBA (*n *= 2632)	Total (*n *= 15,260) PBA (*n *= 10,091) RBA (*n *= 5169) All implants were teardrop-shaped, textured silicone gel	PBA Incision (% devices): Inframammary (91.6%) Peri-areolar (6.1%) Other/unknown (2.3%) Placement: Subglandular (9.3%) Subpectoral (2.3%) Dual plane (88.2%) Other/unknown (0.2%) RBA Incision(% devices): Inframammary (75.3%) Peri-areolar (10.8%) Other/unknown (13.9%) Placement: Subglandular (21.9%) Subpectoral (8%) Dual plane (68.3%) Other/unknown (1.8%)
					Reconstructive (*n *= 9965) PBR (*n *= 7502) RBR (*n *= 2463)	Total (*n *= 16,725) PBR (*n *= 12,644) RBR (*n *= 4081) All implants were teardrop-shaped, textured silicone gel	PBR Incision (% devices): Mastectomy scar (81.9%) Inframammary (16.6%) Other/unknown (1.5%) Placement: Subglandular (1%) Subpectoral (42.6%) Dual plane (52.7%) Other/unknown (3.7%) **RBR** Incision(% devices): Mastectomy scar (67.7%) Inframammary (27.9%) Other/unknown (4.4%) Placement: Subglandular (3.3%) Subpectoral (31.9%) Dual plane (58.3%) Other/unknown (6.5%)
Tirrell et al. (2022) [45]	USA	Retrospective analysis (C)	18/24	143	Cosmetic: BA (6.7%) Reconstructive: BR (93.3%)	Total (*n *= 254; 6.7% BA, 93.3% BR) All implants were silicone gel Texture and shape NR	Incision: Inframammary and transverse (% NR) Placement (% total devices): Pre-pectoral (90.9%) Retro-pectoral (9.1%)
Venkataram et al. (2022) [39]	USA	Prospective observational study (N)	11/16	2088	Cosmetic: PBA	Total (*n *= 4176, 40% textured, 60% smooth) ãType NR	Incision: NR Placement: NR
Wiener (2007) [36]	USA	Retrospective analysis (C)	11/24	1244	Cosmetic: PBA	Total number of implants NR All implants were saline, round and smooth	Incision: Inframammary (reported as “most”) & peri areolar Placement: Submuscular

C: Comparative; N: Non-comparative; NR: Not reported; BA: Breast augmentation; PBA: Primary breast augmentation; RBA: Revision breast augmentation; BR: Breast reconstruction; PBR: Primary breast reconstruction ; RBR: Revision breast reconstruction; AM: Augmentation mastopexy

### Quality Assessment

The MINORS scores indicated that most studies were of moderate quality (Table 1). Common limitations included retrospective designs, lack of unbiased assessment, and insufficient follow-up periods for long-term outcomes like capsular contracture (CC).

### PVP-I Usage

Data about the utilisation of PVP-I and the outcomes in the included studies are summarised in Table 2. All studies reported using PVP-I for breast pocket irrigation, though the concentration varied (4–20%), and in some cases, the exact strength was not specified. The proportion of patients receiving this treatment was also variable (0.6–100%) and was at times omitted. Only two studies [35, 43] specifically mentioned implant immersion in PVP-I. Other antimicrobials, primarily antibiotics, were frequently used in solution with PVP-I. Reported follow-up periods ranged from 6 to 120 months.

**Table 2:** Povidone-iodine implementation and outcomes

Study (Author, Year)	PVP-I Implant immersion and/or pocket irrigation	PVP-I concentration	Additional antimicrobial agents	Follow-up period (measure)	Periprosthetic breast infection rate	Capsular contracture rate	Other outcomes (rate)
Adams et al. (2006) [32]	Pocket irrigation with solution containing PVP-I before the year 2000 (study spanned from 1997 to 2004); unspecified number of patients	NS; presumably 10% *Solution contained 50 ml of unspecified PVP-I concentration in 500 ml of saline	All patients received preoperative intravenous antibiotics (cefazolin or vancomycin/gentamicin for penicillin-allergic patients). Pocket irrigation solution before 2000 also contained cefazolin and gentamicin Implants bathed in TAS prior to insertion (bacitracin, cefazolin and gentamicin)	14 months mean)	BA group: 0/165 (0%) AM group: ≤1/22 (4.5%) BR group: ≤1/63 (1.6%) Rate in PVP-I group NS *Site of infection NS	BA group: 3/165 (1.8%) AM group: 0/22 (0%) BR group: 6/63 (9.5%) Rate in PVP-I group NS *Defined as Baker 3/4	BA group: Total reoperation rate: 7/248 (2.8%) AM group: Total reoperation rate: 4/24 (16.7%) BR group: Total reoperation rate: 6/63 (9.5%) Deflation/rupture: 2/63 (3.2%)
Araco et al. (2007) [43]	63.4% of patients (*n *= 1902) received antiseptics/antibiotics for pocket irrigation; unclear proportions and whether PVP-I was the antiseptic used All implants (100% of patients) were washed with PVP-I solution before insertion	NS	All patients received preoperative intravenous antibiotics (cefuroxime 750 mg or erythromycin 1 g if referred with specific allergies) Some patients received antibiotic pocket irrigation (cefuroxime or gentamicin for patients referred with specific allergies)	Patients were reviewed postoperatively at days 7 and 30 and then at 6 months	Total: 33/3002 (1.1%) Patients receiving unspecified pocket irrigation: 15/1902 (0.8%)	14/3002 (0.5%) Rate in PVP-I group NS *Criteria NS	Total reoperation rate: 47/3002 (1.6%)
Blount et al. (2013) [33]	PVP-I pocket irrigation in 0.6% of patients (*n *= 5)	5%	Bacitracin solution pocket irrigation in 18.5% of patients TAS (bacitracin, cefazolin, and gentamicin) pocket irrigation in 11.7% of patients	14.9 months (mean)	Total: ≤ 6/856 (0.7%) *Site of infection NS Rate in PVP-I group NS	Total: 24/856 (2.8%) Rate in PVP-I group NS *Defined as Baker 3/4	Total complication/ reoperation rate: 158/856 (18.4%) Total implant deflation: 2/856 (0.2%)
Calobrace et al. (2018) [34]	PVP-I pocket irrigation in 20.9% of patients (*n *= 537)	NS	An unspecified proportion of patients received antibiotic pocket irrigation An unspecified proportion of patients received steroid pocket irrigation	10 years (study duration)	NR	Total: 224/2565 patients (8.7%) PVP-I irrigation group: 25/537 implants (4.7%) *Defined as Baker 3/4	NR
Giordano et al. (2013) [35]	Pocket irrigation with solution containing PVP-I in 50% of patients (*n *= 165) Implants were bathed in this solution before insertion (in this group only)	4%	Irrigation solution also contained cefuroxime and gentamicin IV cefuroxime administered intraoperatively	Irrigation group: 22 months (mean)	NR	Total: 11/330 (3.3%) Irrigation group: 1/165 (0.6%) *Defined as Baker 3/4	Seroma in irrigation group: 2/165 (1.2%) Superficial wound infection in irrigation group: 2/165 (1.2%)
McGuire et al. (2017) [44]	Pocket irrigation with PVP-I in: 16.8 % of PBA (*n *= 1695) 20.4% of RBA (*n *= 1054) 20.1% of PBR (*n *= 2542) 22.4% of RBR (*n = *914)	NS	Pocket irrigation using antibiotics in >70% of each group. (unclear if mixed or separate from PI irrigation)	PBA: 4.1 years RBA: 3.7 years PBR: 2.9 years RBR: 3.5 years (mean)	NR	Total: PBA: 2.3% RBA: 4.1% PBR: 3.1% RBR: 4.0% Rate in PVP-I group N *criteria NS	Late seroma in 0.06% of PBA, 0.15% of RBA, 0.06% of PBR, 0.22% of RBR
Tirrell et al. (2022) [45]	Pocket irrigation with solution containing PVP-I in 85% (*n *= 216) of patients	≤ 10% *10% PVP-I was used with saline/lactated Ringer*’*s +/- antibiotics (unclear if combined)	Various antibiotics used in pocket irrigation solution for unspecified patients including bacitracin cefazolin, gentamycin, vancomycin and polymyxin B	296.2 days (mean)	Total 18/254 (7.1%) Rate in PVP-I group NS	NR	Total complication rate: 9.8% Total seroma rate: 2%
Venkataram et al. (2022) [39]	Pocket irrigation with solutions containing PVP-I in an unspecified number of patients (Betadine Triple used before 2000 and in select patients after 2000, 50% Betadine was used in select patients after 2000)	10–20% *Betadine Triple 50–100 ml of PVP-I stock solution in 500ml of saline 5% *50% Betadine	Irrigation solution (Betadine Triple) also contained cefazolin and gentamicin in an unspecified number of patients Some patients received a non-Betadine containing TAB solution (bacitracin, cefazolin, gentamycin)	6 years (mean)	NR	12/2088 (0.6%) Rate in PVP-I group NS *reported using Baker system, unclear which scores classified as CC	NR
Wiener (2007) [36]	Group 1: Betadine pocket irrigation (*n *= 538, 43.2%) Group 2: Saline rinse after Betadine pocket irrigation (*n *= 341, 27.4%) Group 3: Betadine pocket irrigation (*n *= 154, 12.4%) Group 4: Betadine pocket irrigation + placement of Betadine-soaked gauze on nipple-areola complex and incision site (*n *= 211, 17%)	5% *Betadine and saline combined in 1:1 ratio	All patients received perioperative IV cefazolin and postoperative oral cefalexin	G1: 45 months G2: 24 months G3: 11 months G4: 5 months (mean)	Total: ≤2/1244 Rate in each group NS *site of infection NS	G1: 7/538 (1.3%) G2: 14/341 (4.1%) G3: 5/154 (3.2%) G4: 1/211 (0.5%) *Defined as “Grade 2–4*”*	Deflation rates G1: 2/538 (0.4%) G2: 0/341 (0%) G3: 1/154 (0.6%) G4: 0/211 (0%)

“Betadine” when reported was assumed to be 10% PVP-I

PVP-I: Povidone-iodine; NS: Not specified; NR: Not reported; BA: Breast augmentation; PBA: Primary breast augmentation; RBA: Revision breast augmentation; BR: Breast reconstruction; PBR: Primary breast reconstruction; RBR: Revision breast reconstruction; AM: Augmentation mastopexy

### Periprosthetic Infection

Reporting on periprosthetic infection was inconsistent. Four studies did not report these infections, while in three studies, the distinction between superficial wound infections and peri-implant infections was unclear. Only two studies clearly reported peri-implant infection rates, with one [43] observing a 1.1% infection rate in cosmetic procedures and the other [45], 7.1% in mainly reconstructive cases. However, the proportion of patients receiving PVP-I treatment who developed infections was not specified, nor was it clear how many patients who developed infections had received PVP-I treatment.

### Capsular Contracture

Most studies reported CC occurrence and typically defined this as Baker grade 3 or 4. The overall rates ranged from 0.5 to 8.7% in cosmetic and 3.1 to 9.5% in reconstruction procedures, but in the majority, the proportion of these cases exposed to PVP-I was not presented. In the two studies that reported a comparison involving primary breast augmentation, PVP-I-treated patients had lower capsular contracture (CC) rates compared to those who did not receive PVP-I treatment. One study [35], using a 4% concentration showed a statistically significant difference (0.6 vs. 6%, *P *= 0.006), while the other [34] reported a non-significant reduction (4.7 vs. 6.7%, *P *= 0.2916). However, it is important to note that PVP-I was not always the sole antimicrobial irrigation agent in either study and the concentration was not specified in the second.

### Other Outcomes

Other complications such as implant deflation, rupture, and seroma formation were seldom and inconsistently reported. Moreover, even when reported, it was often unclear whether the patients received PVP-I treatment and the concentration used, making fruitful analysis of this measure futile.

## Discussion

The utilisation of PVP-I in implant-based cosmetic and reconstructive breast surgeries, specifically for the irrigation of the breast pocket and bathing of prostheses, currently lacks universally accepted guidelines. This review is primarily focused on how PVP-I concentration affects outcomes such as periprosthetic infection and CC and also considers its influence on implant integrity in the setting of historical concerns about deflation and rupture that have been largely discredited [46–48]. In approaching this research question, a scoping review methodology was chosen over a systematic review, acknowledging the expected and observed heterogeneity and the generally low level of evidence in the existing studies [49].

The search yielded nine articles, encompassing observational studies and retrospective analyses. Randomised controlled trials were absent and none of the studies, based on the MINORS tool, were high quality. The results highlight a significant discrepancy in the use of PVP-I, with concentrations ranging from 4 to 20%. Frequent mixing of PVP-I with several antibiotic types in varying concentrations significantly confounds the results. This inconsistency, coupled with varied surgical techniques, unclear reporting of intervention and outcome data as well as the scope including both cosmetic and reconstructive procedures, complicates the ability to pool data and draw definitive conclusions. The notable lack of studies exclusively dedicated to reconstructive breast surgeries suggests a significant gap in the literature, emphasising the need for targeted research in optimising implant-based breast reconstruction. While the incidences of periprosthetic infections and CC reported were generally low across the studies, the inconsistent reporting and lack of stratification in outcomes measures for patients receiving PVP-I irrigation or implant immersion made it challenging to discern any clear trends or patterns regarding the impact of PVP-I concentration on these outcomes. In the studies that did report the rate of CC specific to the PVP-I irrigation group, it is reassuring that the observed rates were lower than in the cohort not exposed to the intervention. The variability in reported techniques reflects the customised nature of the clinical practice, where surgical approaches are tailored to individual patient needs, but it also underscores the need for more standardised reporting and rigorous research methodologies to better understand the role of PVP-I in these surgical contexts.

Povidone-iodine was first introduced as a surgical antiseptic in the 1950s [50], and after decades of investigation, it is a staple and versatile agent in the reduction of microbial load during procedures [19, 21, 51–53]. A range of PVP-I formulations are used for various purposes however most broadly used for intraoperative irrigation is the 10% aqueous solution most widely known as the name of the commonly used brand Betadine^®^. [54] In the context of breast implant surgery, its use as a pocket irrigant can be tracked back in the literature to the 1980s [55]. It presents a readily accessible, relatively hypoallergenic, and cost-effective option that minimises medication error risk [48, 56]. However, it has been subject to controversy particularly when the substance*’*s contact with implants was deemed a contraindication by the US Food and Drug Administration (FDA) due to concerns of a detrimental effect on implant surface integrity [46, 57]. A considerable number of surgeons objected to this decision and continued to use PVP-I off-label [32, 56]. Over a decade later, numerous studies [47, 58] refuted the claim of PVP-I*’*s damaging effect on the integrity of breast implant shells and consequently, in 2017, the FDA issued a statement revoking the warning against the contact of 10% PVP-I with breast implants. Since then, PVP-I has remained a staple in this context. However, new concerns have emerged, particularly regarding the sterility of the solutions used [59, 60]. Swanson*’*s 2022 article points out that although Betadine solution has been used for implant irrigation for decades, the commonly used 10% bottles carry warnings such as “Antiseptic Nonsterile Solution” and “For External Use Only.” This valid concern warrants greater awareness and necessitates further investigation, as surgeons might unknowingly contaminate breast pockets intraoperatively with Betadine solution. Additionally, the idea that sterilised implants should not require antimicrobial irrigation if adequate sterile technique is used raises questions about the true efficacy of this practice. It might also be worth considering alternative PVP-I-containing products that have been sterilised during their manufacturing process such as a 5% povidone-iodine ophthalmic prep solution [59, 61].

Povidone-iodine is an iodophor (iodine-releasing agent) composed of the compound polyvinylpyrrolidone (PVP) and iodine. When dissolved in water, it exists in equilibrium between the PVP-I complex and free iodine, the latter being responsible for its bactericidal activity. The antimicrobial activity of PVP-I follows a bell curve; it initially increases with its concentration as both the total and free iodine levels rise, reaching a maximum (of free iodine) around a concentration of 0.1% [62, 63]. From this point, the antimicrobial activity, counterintuitively, decreases as the PVP-I concentration increases. Although the total iodine content rises, the free iodine level drops. As a result, PVP-I at a concentration of 1% is a more potent antiseptic than at concentrations of ≥ 10%, a concept that appears to be seldom considered when surgeons prepare irrigation solutions.

The other primary concern with PVP-I*’*s concentration is how it affects adverse effects experienced by patients. Numerous in vivo and in vitro studies have shown PVP-I*’*s toxicity across various tissue types [64–69]. Moreover, the acute absorption of large quantities of free iodine by mucosa can potentially lead to systemic iodine toxicity, especially affecting cardiovascular and renal function [70, 71]. Since these toxic effects are attributed to the iodine component of PVP-I, theoretically, the relationship between concentration and risk should follow the previously mentioned bell-curve pattern, with the highest risk at a concentration of around 0.1%. However, it is important to consider that the common practice of saline rinse after irrigation [32, 35, 36] might minimise these risks. Furthermore, the infrequency of reported cases of iodine toxicity and clinically significant cytotoxicity during breast implant surgery suggests that such events are rare if they occur at all. Like any foreign material introduced into the body, PVP-I pocket irrigation carries a potential risk for allergic reactions and anaphylaxis, as documented in the literature [72]. In such cases, the concentration of the antiseptic should not be a primary concern, as its use should be contraindicated altogether. Alternative antimicrobial irrigation solutions would be preferable in these scenarios.

This study has several limitations. Firstly, the decision to investigate implant-based surgery broadly, rather than focusing specifically on aesthetic or reconstructive procedures, significantly widened the scope and contributed to the observed heterogeneity. Pre-emptive scoping of the literature revealed a notable paucity of relevant primary articles and even when focusing solely on either aesthetic or reconstructive procedures, significant methodological and technical inconsistencies were evident. Consequently, a broad, primarily qualitative scoping review methodology was deemed appropriate.

Among the included studies, the lack of high-quality methodologies undermines the generalisability of any drawable conclusions. Furthermore, the reporting of techniques, parameters, and outcomes in this field often lacks detail. The interchangeable use of the term "Betadine" with PVP-I adds ambiguity and speculation, as Betadine refers to specific branded products rather than the compound itself. These shortcomings limit the feasibility of conducting formal statistical analyses that could provide more robust findings.

Ideally, this topic would be best investigated using a systematic review and meta-analysis study design. However, given the current state of the literature, this approach is impractical and unlikely to yield meaningful results. In the first instance, high-quality studies exploring the efficacy and safety of PVP-I pocket irrigation and implant immersion in implant-based breast surgery are urgently required with a strong emphasis on comprehensive but clear reporting of dose-related outcomes.

## Conclusion

Existing studies report a wide range of PVP-I concentrations, from 4 to 20%, with significant variability in its application and inconsistent reporting of outcomes. Despite its widespread use in breast implant surgeries, current evidence is insufficient to determine the optimal concentration or application methods. Surgeons should remain mindful of concerns regarding the necessity and sterility of commonly used products as well as the counterintuitive effects of PVP-I concentrations on antimicrobial activity within standard ranges. While this review does not establish a direct relationship between PVP-I concentration and implant-based surgery outcomes, it consolidates available evidence and underscores the urgent need for detailed further research to develop evidence-based guidelines. Such efforts are crucial to improving surgical outcomes and enhancing patient care.
